# Induction of intestinal barrier dysfunction in dairy heifers: Evaluation of new serum inflammatory markers and method for quantifying intestinal hyperpermeability

**DOI:** 10.3168/jdsc.2025-0740

**Published:** 2025-06-03

**Authors:** K.E. Vagnoni, E. Lopez-Cruz, M. Carranza, D.B. Vagnoni

**Affiliations:** Department of Animal Science, California Polytechnic State University, San Luis Obispo, CA 93407

## Abstract

Experimental induction of intestinal barrier dysfunction (e.g., inflammation and hyperpermeability) has been shown to induce a systemic inflammatory response and reduce productivity in lactating dairy cows. Because numerous natural situations on-farm (e.g., ruminal acidosis, heat stress, weaning) can impair intestinal barrier function, this is an important phenomenon to study. Therefore, our objective was to induce intestinal barrier dysfunction and evaluate new serum inflammatory markers as well as a new approach to measuring intestinal hyperpermeability. This was accomplished via oral aspirin administration for 21 d in 10-mo-old Holstein and Jersey heifers. Twelve heifers (6 each, Holsteins and Jerseys) were blocked by breed and then randomly assigned (3 heifers per breed) to receive either 0 or 200 mg aspirin/kg BW per day orally. At 0600 h on d 21 of the experiment, urine and blood samples were collected from each animal. Heifers then were dosed orally with gelatin capsules containing 50 g of Co-EDTA using a balling gun. Urine samples were subsequently collected at 1, 3, 6, 8, 12, 18, 24, 30, and 36 h after dosing. Urine samples were analyzed for Co and creatinine, and serum samples were analyzed for the inflammatory markers haptoglobin (Hp), LBP, FABP2, and TNF. Modeling urinary Co:creatinine ratios using a nonlinear function yielded an excellent fit and indicated that urinary Co excretion, a measure of intestinal permeability, was not increased due to aspirin but was higher for Jersey than for Holstein heifers. Also, serum concentrations of Hp and LBP were unaffected, but serum concentrations of FABP2 and TNF were increased due to aspirin administration. Finally, analysis of covariance indicated that serum TNF concentrations were highly correlated with urinary Co excretion. These data suggest that FABP2 and TNF may be valuable additional markers for the study of intestinal barrier dysfunction.

## Highlights

• Oral aspirin administration did not induce intestinal hyperpermeability.
• Oral aspirin administration did appear to induce intestinal inflammation.
• FABP2 appears to be a useful marker of intestinal inflammation.
• Serum TNF appears to be a useful marker of intestinal hyperpermeability.

## Graphical Abstract

**Figure:**
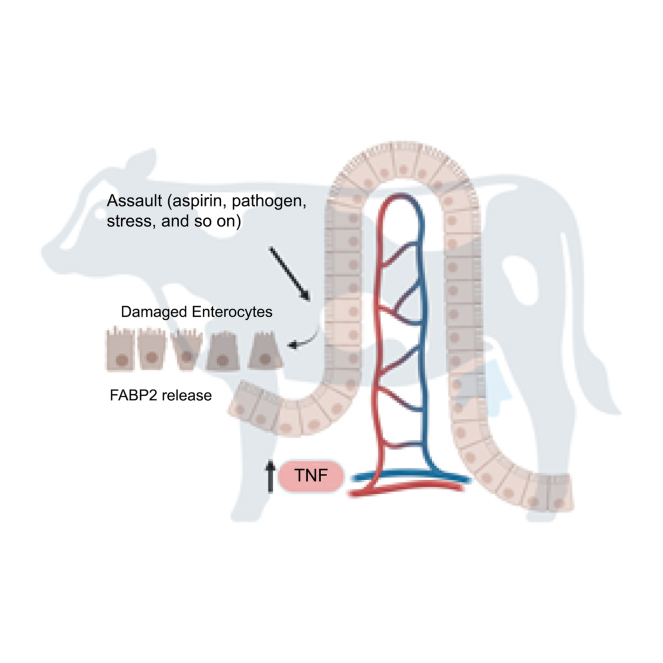
**Summary:** Intestinal barrier dysfunction (e.g., inflammation, hyperpermeability) may arise due to various stressors and results in reduced cow productivity. Determining the best markers of this phenomenon would be useful for the purpose of diagnosis and intervention. To study this, we used oral aspirin administration for 21 days to induce intestinal barrier dysfunction in 10-month-old Holstein and Jersey heifers. Concentrations of serum proteins (as inflammatory markers) and urinary excretion of an indigestible nonmetabolizable marker (as a marker of hyperpermeability) were measured to assess intestinal barrier dysfunction. For serum proteins measured, neither haptoglobin nor serum amyloid A increased due to aspirin treatment. However, both FABP2 and TNF were increased by aspirin, and TNF was also highly correlated to intestinal hyperpermeability. In summary, FABP2 and TNF appear to be promising markers of intestinal barrier dysfunction.

Immune activation and the resultant inflammatory response, triggered by intestinal barrier dysfunction, results in partitioning nutrients away from productive functions (Johnson, 1997; Ceciliani et al., 2012; Bradford et al., 2015). For example, in an inflammatory response to a pathogen, energy that would otherwise be used for growth is used to maintain the integrity of the epithelial lining of the mucosa (the barrier arm of innate immunity) and fuel the response by associated immune cells to the pathogen (Bradford et al., 2015). In addition to a pathogenic insult, heat stress, subacute ruminal acidosis, feed restriction, weaning, and social stress have been shown to negatively affect intestinal barrier integrity, as reviewed by Horst et al. (2021). Reduction in barrier function that results in increased intestinal permeability, specifically the breakdown of tight junctions between epithelial cells, is referred to as “leaky gut” (Chase, 2018; Chase and Kaushik, 2019).

Determining the best markers for intestinal inflammation and permeability is useful for the purpose of diagnosis and intervention, including targeted treatment (Mahapatro et al., 2021). One likely candidate is the proinflammatory marker TNF, which is produced by epithelial cells, fibroblasts, and activated immune cells (Fujisawa et al., 2019). This cytokine has been described as a crucial driver of intestinal inflammation (Mahapatro et al., 2021). In humans, both TNF and IL6 have been shown to be central regulators of inflammatory bowel disease. The role of TNF in intestinal diseases in humans is underscored by the various TNF-targeted therapies used to treat these diseases (Leppkes et al., 2014). Recently, heat-stressed dairy cows were reported to have an increase in plasma TNF (Ruiz-González et al., 2022). This same study showed an increase in the acute phase protein LBP. Increases in LBP and an additional acute phase protein, haptoglobin (**Hp**), have been reported in ketotic cows compared with healthy cows (Abuajamieh et al., 2016). The protein FABP2 is derived from enterocytes, the most abundant epithelial cells in the small and large intestines (Hooper, 2015). Its appearance in circulation in humans indicates damage to mature enterocytes, resulting in leaky gut (Camilleri and Vella, 2022). Therefore, FABP2 has been suggested to be a useful and sensitive marker for early detection of intestinal damage in humans, as it is highly expressed on the tops of the villi, often the initial site of destruction in numerous intestinal diseases (Derikx, 2010). Recently, Ok et al. (2020) determined that FABP2 was a useful biomarker of intestinal damage associated with *Escherichia coli* diarrhea in calves.

A variety of experimental models and methods to induce intestinal barrier dysfunction and measure subsequent responses by cattle have been evaluated. Methods of induction include i.v. administration of a gamma-secretase inhibitor (Kvidera et al., 2017a), oral administration of aspirin (Briggs et al., 2020, 2021), and feed restriction (Kvidera et al., 2017b; Bertens et al., 2024). Intestinal inflammation is typically assessed by measuring serum concentrations of acute phase proteins (Kvidera et al., 2017a; Briggs et al., 2020, 2021; Horst et al., 2020), and intestinal hyperpermeability is typically assessed using measurements of intestinal morphology (Kvidera et al., 2017a,b; Horst et al., 2020) or the appearance of indigestible, nonmetabolizable markers in the plasma or urine (Briggs et al., 2020; Horst et al., 2020; Bertens et al., 2024). Bertens et al. (2024) recently administered the markers Cr-EDTA and Co-EDTA (Udén et al., 1980) ruminally and abomasally, respectively, to determine total-tract and postruminal permeability, in a feed-restriction model of intestinal barrier dysfunction, and made several excellent points. First, measuring plasma marker concentrations at individual time points after dosing could lead to erroneous conclusions due to differences in the time course appearance of markers in blood. Second, although total urine collection is the gold standard, allowing for quantification of marker excretion, it is experimentally restrictive and carries associated risks (e.g., incomplete urine recovery, urinary tract infections). Finally, computing the area under the curve for plasma marker concentrations from serial blood samples taken after dosing does not allow for quantification of marker excretion.

Our objectives were two-fold. First, we evaluated 2 promising inflammatory markers (in addition to acute phase proteins) based on results reported in work on human intestinal barrier dysfunction, namely FABP2 and TNF (Leppkes et al., 2014; Mahapatro et al., 2021; Camilleri and Vella, 2022). Second, we evaluated the quantification of intestinal permeability using sequential spot samples of urine (i.e., without need for total urine collection), using the indigestible, nonmetabolizable marker Co-EDTA. This was facilitated by the fact that urinary creatinine is excreted at a relatively constant rate (29 mg/kg BW per day) in dairy cows (Valadares et al., 1999; Tebbe and Weiss, 2018), which obviates the need for total urine collection, with its attendant risks and restrictions. Details of the associated calculations are provided herein.

Animals were housed at the California Polytechnic State University (San Luis Obispo, CA) Dairy Unit, and animal use was reviewed and approved by the California Polytechnic State University Institutional Animal Care and Use Committee. Based on the work of Briggs et al. (2020, 2021), we used oral aspirin administration (200 mg/kg BW per day) for 21 d to induce intestinal barrier dysfunction. Briggs et al. (2020) observed responses (mean ± SD) of 40.7 ± 20.8 μg/mL for serum amyloid A (**SAA**) and 14.6 ± 8.4 μg/mL for LBP due to aspirin administration. Allowing for a power of 80% and an acceptable type I error risk of 5%, these yield sample size estimates of 4 and 5 animals, respectively. Thus, we enrolled 6 animals per treatment group. Because it appears that, to date, all data on intestinal barrier dysfunction in dairy cattle has been conducted with Holsteins, we included both Holsteins and Jerseys to allow for the evaluation of potential breed differences. Twelve total heifers (6 Jerseys, 10.4 ± 0.3 mo of age, 269 ± 21 kg BW; 6 Holsteins, 10.5 ± 0.9 mo of age, 362 ± 25 kg BW) were enrolled in the experiment. Heifers were blocked by breed, and then 3 animals of each breed were randomly assigned to receive either 0 or 200 mg aspirin/kg BW per day for 21 d. Heifers were housed in a single pen (loose housing, bedded with dried manure solids) with individual locking stanchions and fed a TMR once daily at 0600 h, consisting of grass hay, lactation diet refusals, almond hulls, soybean meal, minerals, and vitamins. The diet contained (DM basis) 13.6% CP, 36.6% ash-free neutral detergent fiber organic matter, and 0.97 Mcal NEG/kg. One-half the daily aspirin dose was administered at each of 0600 h and 1800 h daily. Aspirin (VetOne, product no. V1 501056) was crushed and delivered as a slurry in 200 mL of water using a drenching gun. Authors were not blinded to treatments.

At 0600 h on d 21, a sample of urine (midstream from each heifer by manually stimulating the area immediately ventral to the vulva) and blood (from the coccygeal artery or vein into a serum tube) was collected from each heifer. Thereafter, heifers were dosed orally with gelatin capsules containing 50 g of Co-EDTA (Udén et al., 1980) using a balling gun. Urine samples were subsequently collected at 1, 3, 6, 8, 12, 18, 24, 30, and 36 h following dosing. Blood samples were refrigerated immediately upon collection, centrifuged (2,000 × *g*, 10 min, 5°C), and serum was stored at −20°C. Urine samples were acidified with 6 *N* HCl to pH <3 and stored at −20°C.

Urine samples were analyzed for Co (Sandell, 1959) and creatinine (Oser, 1965). Serum Hp was determined by a colorimetric method based on peroxidase activity (Cooke and Arthington, 2013). Other serum inflammatory markers were assayed by bovine-specific ELISA from MyBioSource, including FABP2 (MBS2609312), TNF (MBS2701332), and LBP (MBS4500695). According to the manufacturer, each of these assays is reported to be free from cross-reactivity with analogs or other factors. For the TNF ELISA, samples were diluted 1:10. The intra-assay CV was <10% and the inter-assay CV was <12% in all cases for all assays.

Urinary Co:creatinine ratios were fitted to the following function (Dhanoa et al., 1985):

$$f(t)=\left[{Ae}_{}^{-k_{1}t}\right]\times \exp\;\left[-{Be}_{}^{-k_{s}t}\right],$$

using a nonlinear mixed effects model via the saemix package of R (Comets et al., 2017). This results in a set of parameter estimates (i.e., A, B, k_1_, k_2_) and a fitted curve for each heifer. In this equation, t represents time, e is Euler's number, and A, B, k_1_, and k_2_ are parameters. Multiplying the area under the resulting curve for each heifer (i.e., [mg Co/mg creatinine] × d; determined by the trapezoidal rule) by the BW (kg) of each heifer and by the daily urinary creatinine excretion (29 mg/kg BW per day; Valadares et al., 1999; Tebbe and Weiss, 2018) results in an estimate of total urinary Co excretion (mg/d). Urinary Co excretion and serum marker concentrations were analyzed by ANOVA using the lm function of the stats package (R Core Team, 2023) to determine the effects of aspirin, breed, and the interaction of aspirin with breed. The relationship of each serum marker with urinary Co excretion was analyzed via analysis of covariance (**ANCOVA**); the model included the fixed effects of aspirin and breed, the continuous effect of urinary Co excretion, and all interactions. Starting with the highest-order term, nonsignificant (*P* > 0.05) terms were removed from the model in a stepwise manner until only significant (*P* ≤ 0.05) terms remained.

Graphical evaluation of observed versus fitted values of urinary Co:creatinine ratios (Figure 1) indicated an excellent fit. Also, formal testing of the adequacy of the model fit by evaluating the normalized prediction distribution errors (Brendel et al., 2006; Comets et al., 2008) indicated that the distribution of residuals did not differ (*P* ≥ 0.4) from a normal distribution (via Shapiro-Wilks test) with a mean of 0 (Wilcoxon signed-rank test) and variance of 1 (Fisher variance test). Moreover, overall mean urinary Co excretion (0.25 g/d) represented 3.6% of the Co dose (i.e., 50 g of the Co-EDTA marker corresponds to 6.9 g of elemental Co), which is in good agreement with the results obtained (“upwards of 3%”) from total urine collections in the original work reporting the development of the marker (Udén et al., 1980). Collectively, these results indicate successful application of the present methodology to assess urinary Co excretion. In contrast to expected results (Briggs et al., 2020, 2021), urinary Co excretion was unaffected (*P* = 0.75) by aspirin administration but was higher (*P* = 0.007) for Jerseys than for Holsteins (Table 1).

**Figure 1:**
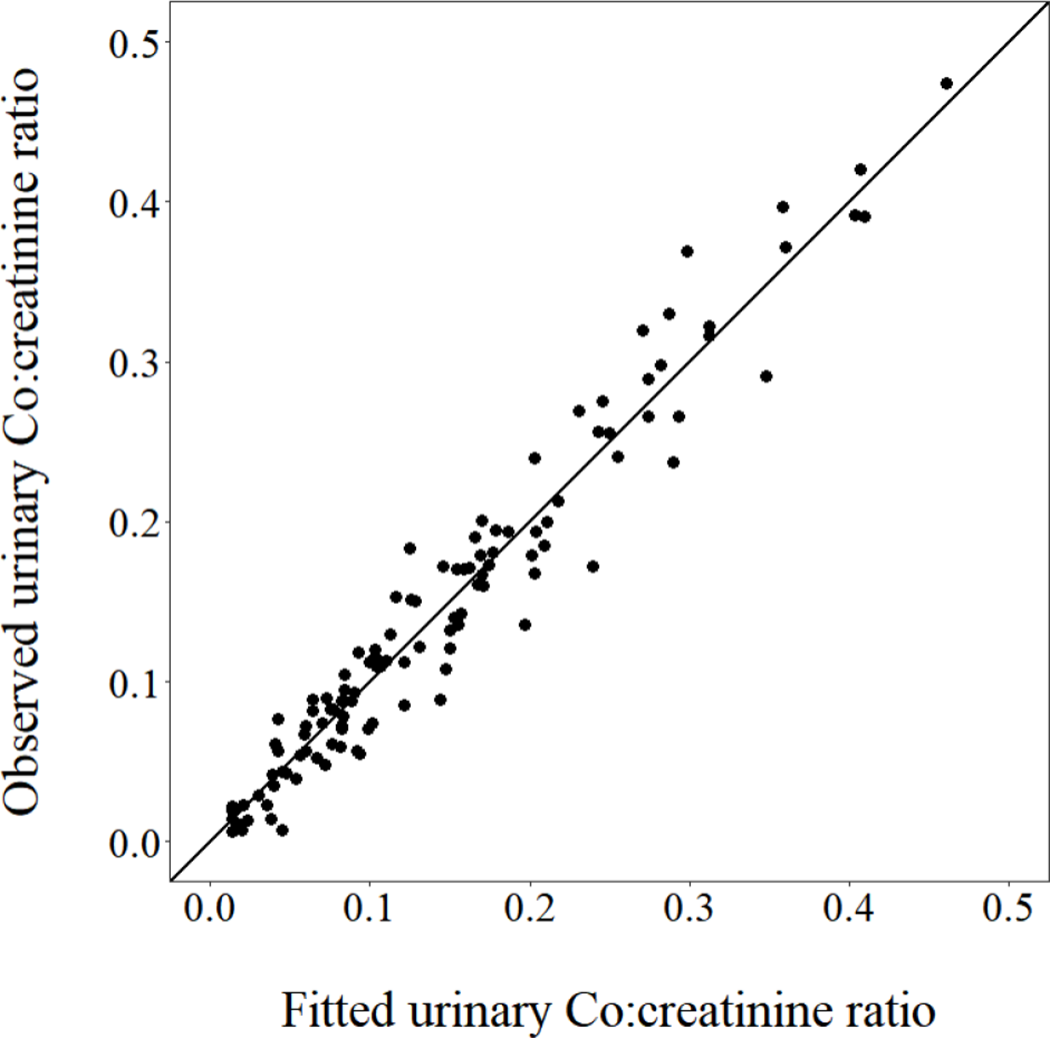
Observed versus fitted urinary Co:creatinine ratios (mg/mg) from the model of Dhanoa et al. (1985), with the line of x = y.

**Table 1:** Concentrations of serum inflammatory markers and urinary Co excretion in heifers (n = 3 animals/group)^1^

Variable	Group				SEM	*P*-value		
	H0	H200	J0	J200		Aspirin	Breed	Aspirin × breed
Urinary Co excretion, mg/d	210	239	292	274	16.5	0.75	0.007	0.20
Serum Hp, μg/mL	356	369	407	443	118	0.84	0.61	0.92
Serum LBP, ng/mL	394	456	325	387	41.5	0.20	0.15	0.13
Serum FABP2, pg/mL	120	282	200	362	47.5	0.03	0.23	1.0
Serum TNF, pg/mL	41.2	103.2	85.3	147.3	22.4	0.05	0.14	0.59

1 H0 = Holsteins, 0 mg aspirin; H200 = Holsteins, 200 mg aspirin/kg BW per day; J0 = Jerseys, 0 mg aspirin; J200 = Jerseys, 200 mg aspirin/kg BW per day.

Serum Hp and LBP concentrations were unaffected (*P* ≥ 0.13) by aspirin, breed, or the interaction of aspirin with breed (Table 1). Both serum FABP2 (*P* = 0.03) and TNF (*P* = 0.05) were increased by aspirin treatment (Table 1). Also, ANCOVA indicated that serum TNF (*P* = 0.025), but not other markers (*P* ≥ 0.7), increased with increasing urinary Co excretion. Additionally, for TNF, there was no interaction of urinary Co excretion with either breed or aspirin treatment (*P* ≥ 0.17), indicating a homogeneity of slopes for the relationship between serum TNF and urinary Co excretion among all treatment groups (Figure 2).

**Figure 2:**
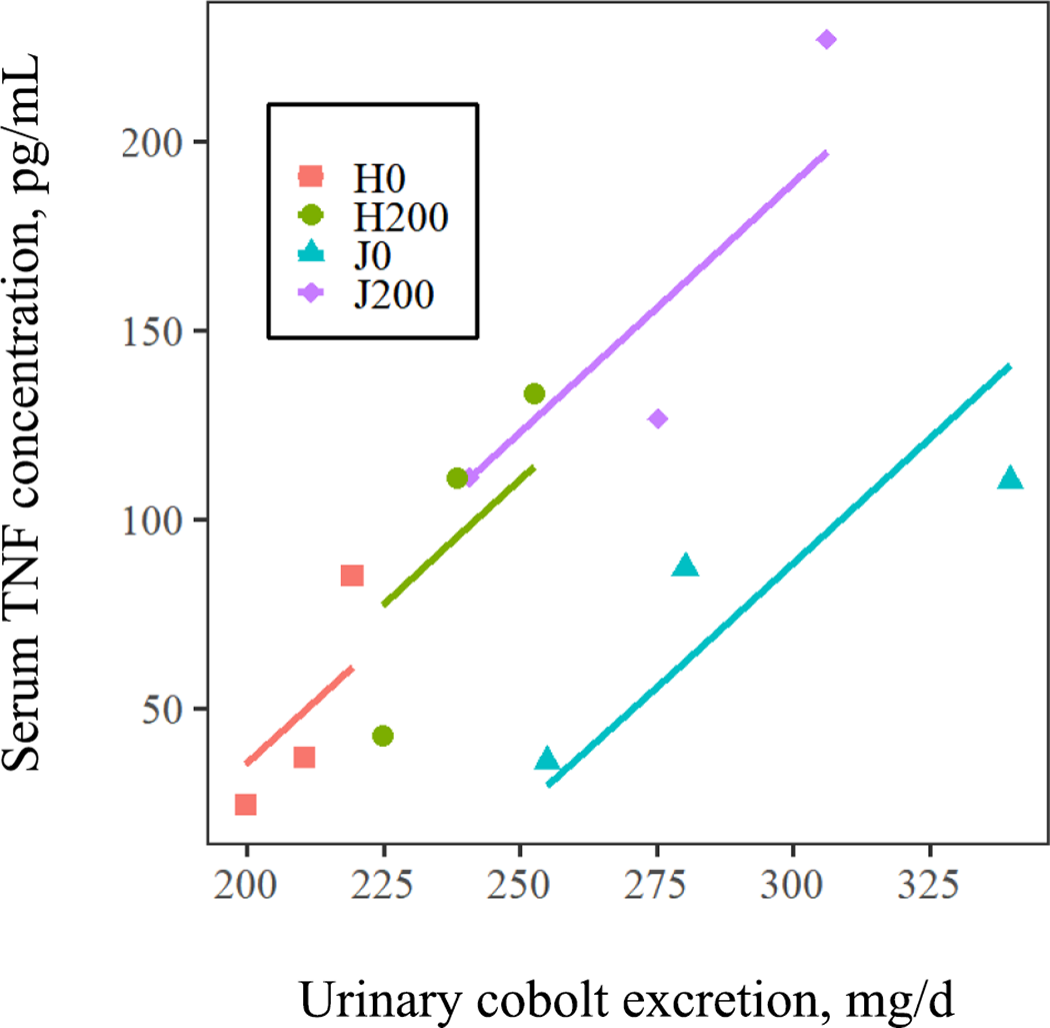
Relationship between serum TNF concentrations and urinary Co excretion for Holstein (H) and Jersey (J) heifers receiving either 0 or 200 mg aspirin/kg BW per day. ANCOVA indicated a linear relationship (*P* = 0.025) between serum TNF and urinary Co excretion but no interactions (*P* ≥ 0.17) of breed or aspirin with urinary Co excretion.

We have been able to find 6 previous reports in cattle where both intestinal barrier dysfunction has been successfully induced, as measured by either intestinal morphology (Kvidera et al., 2017a,b) or increased appearance of Cr in the blood or urine following oral Cr-EDTA administration (Briggs et al., 2020, 2021; Horst et al., 2020; Goetz et al., 2024), and the major acute phase proteins (Hp, SAA, and LBP) were determined. Significant (*P* < 0.05) increases for Hp, SAA, and LBP were found in 2, 4, and 3 of the studies, respectively. Thus, for any given marker, there is approximately a 50% chance of detecting an inflammatory response in the confirmed presence of intestinal barrier dysfunction. Because direct assessment of intestinal barrier dysfunction (e.g., measuring intestinal morphology or recovery of an indigestible and nonmetabolizable marker) is extremely labor intensive, availability of an easily accessible and more reliable marker of this disorder would be beneficial.

We are unaware of any reports of evaluating FABP2 in response to the induction of intestinal barrier dysfunction in cattle. Because the acute phase proteins are not specific to intestinal inflammation, FABP2 would theoretically have the advantage of being much more specific to intestinal barrier dysfunction. This protein is highly expressed in cells present on the tops of villi and is primarily limited to mature enterocytes of the small and large intestines (Derikx, 2010). It has been suggested that FABP2 detected in human circulation (plasma) is a likely candidate for measuring early stages of intestinal damage, because the tops of villi are the initial site of destruction in numerous intestinal diseases and FABP2 is found in low amounts in the blood stream and is cleared rapidly by the kidneys in humans (Derikx, 2010). In calves, Ok et al. (2020) determined that FABP2 was a useful biomarker of intestinal damage associated with *E. coli* diarrhea. In the current study, serum FABP2 increased significantly in response to aspirin, but it was not significantly correlated with urinary Co excretion. This suggests that circulating FABP2 may be a useful marker for intestinal inflammation but not necessarily hyperpermeability.

We are aware of only 1 report of serum TNF concentrations in the confirmed presence of intestinal barrier dysfunction in cattle (Goetz et al., 2024), and it was reported to be below the detectable limits of the assay, which were not reported. In the present study we had to dilute serum samples to reduce TNF concentrations to get them on scale with the standard curve. Being a proinflammatory cytokine, TNF controls multiple cellular processes, including those of intestinal epithelial cells, such as their production of inflammatory cytokines and their proliferation, survival, and death (Leppkes et al., 2014). Intestinal mucus secretion and constitution, both of which are important factors in innate immunity, are also modulated by TNF. The interaction between immune cells and intestinal epithelial cells is regulated by TNF. Also, TNF has been shown to be the primary proinflammatory cytokine involved in the breakdown of tight junctions and subsequent intestinal hyperpermeability (Chase, 2018). Along with immune cells and stromal cells, intestinal epithelial cells are a source of TNF (Roulis et al., 2011; Chase and Kaushik, 2019), and they also express TNF receptors (Mizoguchi et al., 2002). Although TNF contributes to regeneration of the epithelial lining by supporting intestinal epithelial cell migration and proliferation (Leppkes et al., 2014), acute exogenous doses of TNF lead to extensive epithelial death (Roulis et al., 2011). Targeting TNF may prove useful in cases of intestinal inflammation.

Although this is only one study, it does indicate the potential for 2 serum markers, FABP2 and TNF, to provide enhanced specificity, sensitivity, or both, over typical markers employed in the study of intestinal barrier dysfunction. We found significant responses in each of these markers to our induction protocol, as well as a strong correlation between TNF and intestinal permeability. As mentioned previously, results reported in the literature on acute phase protein response to intestinal barrier dysfunction are inconsistent, and we failed to detect any response in Hp or LBP. However, it must be acknowledged that the duration of our induction protocol (21 d) was much longer than typical feed restriction or heat stress models (i.e., 5–7 d), which may confound comparisons between our work and previously published work. The ability to quantify urinary marker (e.g., Co, Cr) excretion without the need for total urine collections (i.e., based on sequential spot urine samples combined with appropriate modeling of the data) allows for increased flexibility in measuring intestinal hyperpermeability in dairy cattle. For example, cases of spontaneous cases of intestinal hyperpermeability (e.g., during weaning, ruminal acidosis) could be confirmed using this technique, and then TNF and FABP2 could be evaluated simultaneously with, for example acute phase proteins, to verify the suitability of the various markers.
